# Comparative Analysis of Treatment Outcomes: Modified Ulnar Gutter Slab vs. Sugar Tong Slab for Distal Radioulnar Joint Instability Following Triangular Fibrocartilage Complex Repair

**DOI:** 10.3390/jcm12206574

**Published:** 2023-10-17

**Authors:** Tulyapruek Tawonsawatruk, Pheeraphat Phoophiboon, Thepparat Kanchanathepsak, Panithan Tuntiyatorn

**Affiliations:** 1 Department of Orthopaedics, Faculty of Medicine, Ramathibodi Hospital, Mahidol University, Bangkok 10400, Thailand; tulyapruek.tao@mahidol.ac.th (T.T.); pheeraphat.pho@mahidol.ac.th (P.P.); thepparat.kan@mahidol.ac.th (T.K.); 2 Chakri Naruebodindra Medical Institute, Faculty of Medicine, Ramathibodi Hospital, Mahidol University, Samut Prakan 10540, Thailand

**Keywords:** distal radioulnar joint, DRUJ instability, TFCC, triangular fibrocartilage complex, modified ulnar gutter slab, sugar tong slab, post-operative immobilization

## Abstract

The standard treatment for distal radioulnar joint (DRUJ) instability involves repairing the triangular fibrocartilage complex (TFCC) and immobilizing the joint with a sugar tong slab, but this can cause elbow stiffness. To address this, a modified ulnar gutter slab was designed to enhance elbow mobility during immobilization. A prospective randomized controlled trial was conducted on 23 DRUJ instability patients who underwent arthroscopic TFCC repair. Two post-operative splinting techniques were compared: the modified ulnar gutter slab and the sugar tong slab. The assessment included the Disabilities of Arm, Shoulder, and Hand (DASH) score; elbow, forearm, and wrist range of motion (ROM); post-operative DRUJ stability; and complications. DASH scores at 4 and 6 weeks were not significantly different. However, the modified ulnar gutter slab improved elbow extension range of motion at 4 weeks (extension lag: 20.0 vs. 6.5 in the sugar tong group) (*p* = 0.011). Post-operative DRUJ stability was comparable between the two groups. Notably, one patient in the sugar tong slab group experienced complex regional pain syndrome (CRPS). The modified ulnar gutter slab offers a post-operative alternative after TFCC repair. It effectively immobilizes forearm and wrist motion while enhancing elbow mobility, potentially reducing post-operative elbow stiffness.

## 1. Introduction

Distal radioulnar joint (DRUJ) instability is a frequently encountered issue in wrist injuries. This condition results from damage to the stabilizing structure of the DRUJ, known as the triangular fibrocartilage complex (TFCC) [1]. It can manifest as an isolated injury [2] or occur in conjunction with forearm fractures, particularly involving the distal radius [3,4,5]. This pathology leads to wrist pain and instability symptoms, significantly affecting a patient’s wrist function in daily life activities [6]. The primary biomechanical change following TFCC disruption is the unstable movement between the articulating surfaces of the DRUJ, where the ulnar head may subluxate on the sigmoid notch of the distal radius [7,8]. Without proper management, instability can progress to distal radioulnar joint arthritis [9,10].

Currently, both conservative immobilization and surgical treatment can be chosen after joint reduction in isolated DRUJ instability and DRUJ instability with concomitant distal radius fractures [1,11]. Surgical treatment for DRUJ instability can be performed either via open or arthroscopic repair of the TFCC [11,12]. Both methods boast success rates exceeding 80 percent, with no significant difference between the two techniques [13,14]. As part of the standard post-operative protocol, the repaired site necessitates a splint to protect the patient’s wrist and limit forearm rotation for a period of 4 weeks, typically using a sugar tong slab, also known as a U-slab. This splint aims to restrict forearm rotation, wrist flexion–extension, and radial-ulnar deviation to prevent excess stress on the healing TFCC [11,13,15]. However, a major drawback of this post-operative splint is the development of elbow stiffness. Due to its design, aside from controlling the wrist, the splint imposes restrictions on elbow movement for an extended duration. This side effect hampers daily activities both during splint wear and even after its removal [16]. In some cases, patients require additional time for elbow physical therapy to address this issue.

In this study, the modified ulnar gutter slab was introduced to allow the flexion–extension movement of the elbow while simultaneously immobilizing the radioulnar joint. We hypothesized that this modified ulnar gutter slab would offer better elbow motion and functional outcomes compared to the sugar tong slab. Therefore, the objective of this study was to compare the clinical and functional results between two different post-operative splinting methods in patients with DRUJ instability who had undergone arthroscopic TFCC repair: the modified ulnar gutter slab and the sugar tong slab.

## 2. Methods

Following approval from the Institutional Review Board of Ramathibodi Hospital, Mahidol University (COA.MURA2020/1724), this study was conducted within the orthopedic department at Ramathibodi Hospital. It involved a prospective, double-blind, randomized controlled trial. The inclusion criteria encompassed patients with acute traumatic DRUJ instability who had undergone TFCC repair within 12 weeks after the injury [17]. This study included patients with either isolated traumatic DRUJ injuries or those with DRUJ injuries combined with associated fractures, specifically distal end radius fractures, Galeazzi fractures, and Essex-Lopresti fractures. Excluded from this study were patients with a history of fracture or DRUJ instability treated conservatively, those with multiple injuries, open fractures, prior hand, wrist, or elbow abnormalities, as well as individuals who declined or withdrew from participation.

To confirm the diagnosis of DRUJ instability in all participants, clinical tests such as the piano key sign and arthroscopic wrist examination were employed. In patients with concomitant fracture, TFCC was addressed separately from the fracture. Demographic information, including age, gender, height, weight, body mass index (BMI), dominant arm, and type of injury, was collected. All patients underwent arthroscopic foveal repair of the TFCC [18], with or without fracture fixation, depending on the presence or absence of a fracture. Subsequently, patients underwent post-operative wrist immobilization in a neutral position for four weeks, after which the splint was removed. Follow-up appointments were scheduled 2, 4, and 6 weeks following the operation.

Based on the previous report on the range of motion on different types of forearm immobilization [19], a sample size of sixteen patients was calculated to be necessary to provide >80% power to detect a minimum difference of 28 in the range of forearm rotation among different types of immobilization with 2- sided significant level of 0.05, assuming a standard deviation of 20. Considering a possible dropout rate of 20%, 20 patients in total were required (8 patients per group).

The randomization of each type of slab was conducted using STATA 15.0 software provided by Stata Corp in College Station, TX, USA. The immobilization procedures were sealed in envelopes and organized based on the study participants’ order. These envelopes remained sealed until after the surgical procedure to ensure that the operating physicians remained unaware of the treatment allocation. During the follow-up, the outcome assessor was also kept blinded to the data.

At the two-week mark, an appointment is scheduled to examine the surgical wound and assess potential complications due to splinting, such as the presence of pressure sores or ulnar nerve compression.

**Figure 1:**
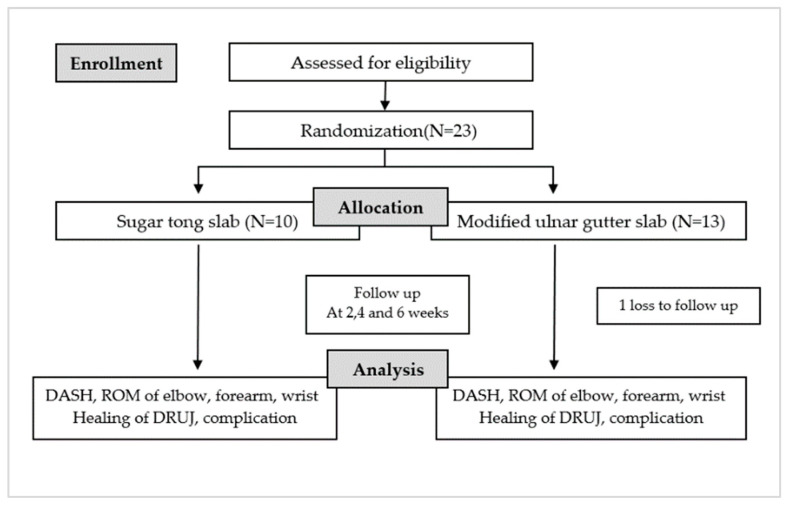
Flowchart of the study protocol.

At the end of the fourth and sixth weeks, patients are asked to complete a questionnaire regarding their quality of life while using the injured arm with the splint, assessed using a DASH score. The clinical examination includes evaluating the angles of the elbow (flexion and extension) and wrist (pronation, supination, flexion, and extension), and performing a piano key test. Additionally, a wrist radiograph is assessed, and all post-operative complications are documented (Figure 1).

**Figure 2:**
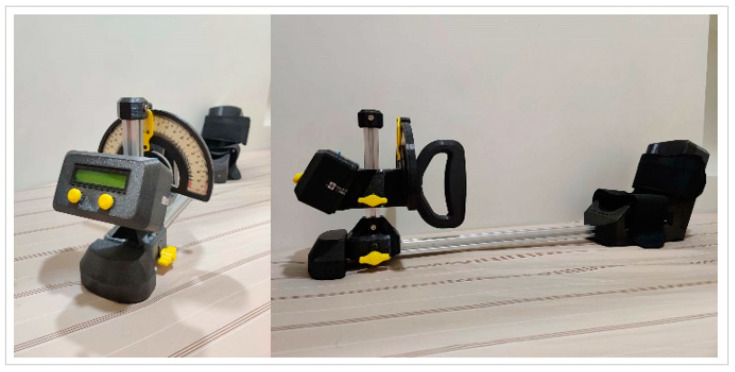
The modified digital goniometer for measuring the forearm rotation.

We used a standard goniometer to measure the range of motion and the angle at the elbow joint. The pivot point was positioned at the midpoint of the joint, and then, the angle between the arm and forearm axis was measured. For the measurement of forearm supination and pronation angles, a modified digital goniometer was utilized (Figure 2).

The post-operative assessment of DRUJ (Distal Radioulnar Joint) stability involved two key evaluations. First, the clinical examination included the piano key test, wherein pressure was applied to the head of the ulna bone while the forearm was in a pronated position. If DRUJ instability was present, the ulna head could be depressed and would automatically rebound after pressure release, indicating a positive test result [20]. Second, the radiographic examination was employed to assess DRUJ stability by measuring the displacement of the distal ulnar head relative to the dorsal rim of the distal radius in a lateral view of the wrist radiograph. Displacement equal to or greater than 5 mm was considered indicative of DRUJ instability [21].

**Figure 3:**
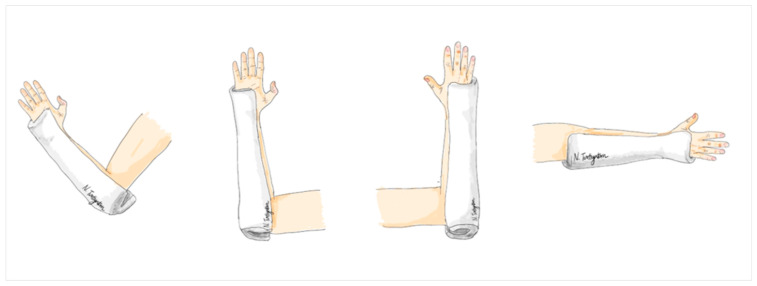
The modified ulnar gutter slab.

The modified ulnar gutter splint is a specific type of splint applied to the ulnar side of the forearm. Its length extends from the distal end, reaching the palmar crease, to the proximal end, which covers the elbow and extends about 2 inches beyond the olecranon to create a posterior socket for elbow extension. Here, the splint was custom molded to fit the forearm, with special attention to the proximal and distal ends between the radius and ulna bones. It was constructed using a 6-inch plaster of Paris (POP) splint roll with a thickness of 8 layers, covering all plaster surfaces with 100% cotton webril padding to prevent skin irritation, abrasion, and excoriation. The width of the splint covered approximately 2/3 of the forearm’s width at both the proximal and distal ends, allowing for gentle elbow flexion and extension in this treatment group (Figure 3).

**Figure 4:**
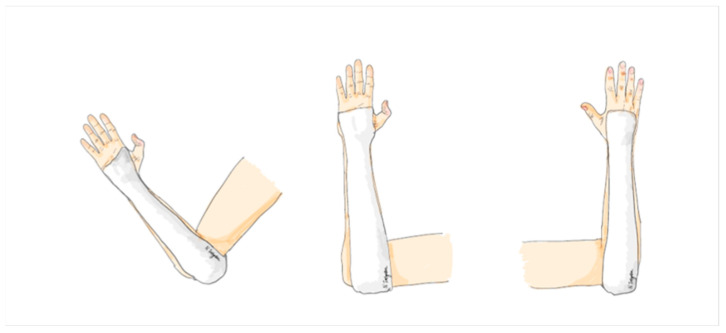
The sugar tong arm slab.

The application of a sugar tong splint involves using a soft splint that starts at the distal palmar crease and extends along the volar aspect of the forearm, wrapping around the posterior elbow in a 90-degree elbow flexion position, then covering the dorsal aspect of the forearm to the dorsum of the hand, and finally ending proximal to the metacarpophalangeal joint. This splint is custom molded to fit the forearm, with careful attention to the proximal and distal radioulnar joint. A 4-inch POP splint roll is used with a thickness of 8 layers, covered all plaster surface with 100% cotton webril padding, and the width of the splint covers approximately 4/5 of the forearm’s width in both the anterior and posterior aspects (Figure 4).

## 3. Statistical Analysis

Data analysis was performed using SPSS Inc., Version 18.0 (PASW Statistics, Released 2009). Continuous variables were expressed as means, while categorical variables were presented as percentages. For demographic data, we compare continuous data between groups. The independent t-test for normally distributed data and the Mann–Whitney U test for non-normally distributed data were utilized. For binary or categorical data, a chi-square test was applied, and appropriate tests were employed depending on whether the data followed normal or non-normal distribution patterns. For the treatment comparison, two-way ANOVA was applied, followed by the Bonferroni test. A *p*-value less than 0.05 was considered to be of statistical significance.

## 4. Results

From October 2020 to October 2022, 23 patients were enrolled, 10 males and 13 females, with an average age of 46 years. Approximately 50% of these patients had an injury in their dominant arm. They were categorized into two groups based on their specific injuries: traumatic TFCC injury (8 participants) and distal end radius fracture with DRUJ instability (14 participants). In the period of study, no cases of Galeazzi fractures and Essex-Lopresti fractures were presented. Out of the total participants, 10 were randomly assigned to wear a sugar tong slab, while 13 were randomly assigned to wear a modified ulnar gutter slab. Only 1 participant was lost to follow-up, leaving 22 participants who were monitored until the end of the 6-week protocol period following surgery. No statistical difference was present in the demographic data between the two groups. (*p* < 0.05) (Table 1 and Figure 1).

The functional outcomes, as measured using the DASH score, showed no significant differences between both groups at 4 weeks and 6 weeks post-surgery. Specifically, the mean DASH score at 4 weeks after surgery for the sugar tong slab group was 59.0, and for the modified ulnar gutter slab group, it was 45.3. Similarly, the mean DASH score at 6 weeks post-surgery for the sugar tong slab group was 42.6, while for the modified ulnar gutter slab group, it was 44.6 (*p* = 0.295) (Table 2).

At 4 weeks following surgery, a statistically significant difference was observed in the occurrence of elbow joint stiffness between the sugar tong slab and modified ulnar gutter slab groups. The extension lag of the elbow joint measured 20.0 degrees for the sugar tong slab group and 6.5 degrees for the modified ulnar gutter slab group (*p* = 0.011). The flexion of the elbow joint measured 132.5 degrees for the sugar tong slab group and 127.5 degrees for the modified ulnar gutter slab group (*p* = 0.048). However, there were no statistically significant differences between the two groups in terms of elbow flexion and extension lag at 6 weeks post-surgery, and no statistically significant differences between the two groups in terms of forearm supination and pronation, wrist flexion, and extension range of motion at both 4 and 6 weeks post-surgery (Table 2).

Regarding the healing of DRUJ, no statistically significant differences were noted between the two groups, as assessed by the piano key test and dorsal displacement of the ulnar head on the lateral view of the wrist radiograph. It is worth mentioning that one patient in the sugar tong slab group experienced a post-operative complication, specifically identified as complex regional pain syndrome (CRPS) (Table 3).

## 5. Discussion

In contemporary orthopedic treatments for forearm ring structure injuries requiring the immobilization of forearm rotation [22,23,24], a noteworthy dilemma arises between the perspectives of orthopedic surgeons and patients. Patients typically prefer the shortest possible splint, such as a short arm slab, short arm cast, or ulnar gutter slab, while orthopedic surgeons often opt for complete forearm stabilization through options like long arm slabs, long arm casts, or sugar tong slabs. This ongoing debate revolves around the choice between allowing free elbow movement and restricting it [25,26,27,28], as it has implications for surgical outcomes and patient comfort. Prolonged elbow restriction can potentially hinder a patient’s activity and lead to discomfort.

To address this issue, the recent literature on post-operative care for arthroscopic TFCC repair procedures has suggested alternatives to standard elbow-restricted splints. For instance, Lerma et al. recommended starting with a brachial splint that permits elbow flexion in a neutral forearm position using a Muenster-type orthosis for 3–4 weeks. Subsequently, the orthosis can be replaced with a forearm brace to allow elbow mobility, combined with isometric exercises over a two-week period [29].

Additionally, a study by Jung et al. explored the effectiveness of short arm casts versus long arm casts in a semi-supination position. The results indicated that both groups exhibited improvement in all clinical outcome parameters with no significant postoperative differences. However, it is noteworthy that the disability associated with the long arm cast on the dominant hand was significantly higher [28].

The modified ulnar gutter slab represents an innovative splinting approach not previously employed. Its core objective is to immobilize the rotational movement of the forearm and wrist joints while safeguarding the healing process of the repaired TFCC. Notably, it allows for full flexion and extension of the elbow concurrently. Conversely, the use of a sugar tong slab achieves the desired wrist and forearm immobilization but also restricts the elbow joint, potentially causing discomfort and an increased likelihood of joint stiffness [30,31] compared to patients utilizing the modified ulnar gutter slab.

In our study, we observed a statistically significant reduction in the degree of elbow extension lag in patients using the modified ulnar gutter slab. This finding suggests a potential reduction in the need for extensive physical therapy to regain elbow extension motion. Moreover, our data revealed that the group utilizing the modified ulnar gutter slab performed comparably to the standard treatment group using the sugar tong slab, as assessed using functional scores and the range of motion for forearm supination and pronation and wrist flexion and extension.

Regarding TFCC healing, no significant differences were detected between the two groups, and no complications were reported in the modified ulnar gutter slab group. Conversely, the sugar tong slab group experienced cases of complex regional pain syndrome, which is associated with joint stiffness and tight ligaments or tendons. Therefore, our findings suggest that the modified ulnar gutter slab offers advantages in terms of elbow extension lag and overall comfort compared to the traditional sugar tong slab without compromising functional outcomes or TFCC healing.

The outcomes of elbow immobilization are influenced by three key factors: (1) the center of rotation of the ulnohumeral joint, (2) the direction of the splint in relation to the center of rotation, and (3) the involvement of the radioulnar joint (proximal, intermediate, and distal parts). In the realm of designing anti-rotational movement splints for forearm rotation, three classic techniques exist: (1) the sugar tong splint, (2) the long arm splint, and (3) the Muenster splint [27]. Both the sugar tong and long arm splints effectively restrict forearm rotation by immobilizing the entire radioulnar joint. However, they have the drawback of wrapping around the posterior side of the ulnohumeral joint, limiting flexion and extension elbow movements. The Muenster splint offers some allowance for active elbow flexion and extension while still restricting a significant portion of the elbow’s movement arc. Nevertheless, its design crosses anterior to the ulnohumeral joint, further limiting elbow flexion–extension [25,27,32,33]. Therefore, the introduction of the modified ulnar gutter splint, which immobilizes the radioulnar joint without crossing the ulnohumeral joint’s movement axis and without extending proximally to the epicondyles, may provide a solution [33]. Additionally, this splint has the potential to control the proximal radioulnar joint in both elbow flexion and extension positions through its proximal olecranon extension component. However, it is worth noting that the bulkiness of this proximal extension may pose challenges for patients in maintaining their elbow within a standard arm sling.

The strengths of this study include its randomized, double-blind, controlled trial design, ensuring a rigorous approach to data collection and analysis. Both study groups shared similarities in factors such as age, gender, BMI, and type of injury.

Nonetheless, this study does have limitations. The sample size was relatively small, but despite this, statistical significance was achieved in terms of reduced extension lag in the modified ulnar gutter slab group, and we found that there is more than 80% statistical power. Another limitation was the relatively short follow-up duration. However, it is important to note that stability after repair was confirmed, and healing was observed. Further follow-up after surgery could provide insights into the long-term trends in the functional outcomes of the elbow joint, which may require ongoing treatment.

## 6. Conclusions

The modified ulnar gutter splint presents a straightforward and accessible post-operative immobilization option for patients who have undergone arthroscopic TFCC repair surgery. This splint effectively manages wrist, forearm, and elbow motion throughout the TFCC healing process. Not only does it minimize the risk of elbow stiffness following splint removal, but it also reduces limitations on elbow motion while the splint is worn.

## Figures and Tables

**Table 1:** Demographic data.

	Sugar Tong Slab (N = 10)	Modified Ulnar Gutter Slab (N = 12)
Age (year), mean (SD)	47 (14)	45 (19)
Gender (%)		
Male	5 (50)	4 (33)
Female	5 (50)	8 (67)
BMI (mean)	24.8	26.7
Type of injury (%)		
Isolated traumatic DRUJ instability	3 (30)	5 (41.6)
Distal radius fracture with DRUJ instability	7 (70)	7 (58.3)
Galeazzi fracture with DRUJ instability	0	0
Essex-Lopresti fracture with DRUJ instability	0	0
Dominant hand (%)		
Right	8 (80)	6 (41.6)
Left	2 (20)	7 (58.3)
Side of operation (%)		
Right	4 (40)	11 (91.6)
Left	6 (60)	1 (8.4)

**Table 2:** Post-operative outcomes.

	Sugar Tong Slab	Modified Ulnar Gutter Slab	*p*-Value between 2 Groups
DASH score			
4 weeks	59.0 ± 14.0	45.3 ± 19.5	0.295
6 weeks	42.6 ± 18.5	44.6 ± 19.7	
Range of motion (degree)			
Elbow flexion			
4 weeks *	132.5 ± 8.6	127.5 ± 6.2	0.048 *
6 weeks	131.6 ± 8.6	127.5 ± 6.2	
Extension lag			
4 weeks *	20.0 ± 17.6	6.5 ± 7.9	0.011 *
6 weeks	5.5 ± 5.8	2.9 ± 4.5	
Forearm supination			
4 weeks	32.0 ± 19.0	35.4 ± 23.6	0.394
6 weeks	61.1 ± 18.3	68.1 ± 17.9	
Forearm pronation			
4 weeks	36.5 ± 16.2	39.4 ± 27.3	0.732
6 weeks	57.2 ± 15.2	58.7 ± 21.6	
Wrist flexion			
4 weeks	28.0 ± 6.3	20.9 ± 10.9	0.230
6 weeks	43.3 ± 15.8	39.1 ± 22.3	
Wrist extension			
4 weeks	11.5 ± 11.0	15.3 ± 11.8	0.654
6 weeks	42.7 ± 15.8	42.6 ± 15.0	

* Statistical significance by post hoc analysis.

**Table 3:** Post-operative stability of the DRUJ and the complication.

	Sugar Tong Slab (N = 10)	Modified Ulnar Gutter Slab (N = 12)
Piano key test		
Positive	0	0
Negative	10	12
Ulnar displacement ≥ 5 mm.		
Positive	0	0
Negative	10	12
Complication		
Positive *	1	0
Negative	9	12

* Complex regional pain syndrome (CRPS).

## Data Availability

Data available on request due to restrictions due to privacy in a small randomized controlled trial and ethical limitations.

## Abbreviations

BMI	Body mass index
CRPS	Complex regional pain syndrome
DASH	Disabilities of Arm, Shoulder, and Hand
DRUJ	Distal radioulnar joint
ROM	Range of motion
TFCC	Triangular fibrocartilage complex
