# Correction: Tawonsawatruk et al. Comparative Analysis of Treatment Outcomes: Modified Ulnar Gutter Slab vs. Sugar Tong Slab for Distal Radioulnar Joint Instability Following Triangular Fibrocartilage Complex Repair. *J. Clin. Med.* 2023, *12*, 6574

**DOI:** 10.3390/jcm14072455

**Published:** 2025-04-03

**Authors:** Tulyapruek Tawonsawatruk, Pheeraphat Phoophiboon, Thepparat Kanchanathepsak, Panithan Tuntiyatorn

**Affiliations:** 1 Department of Orthopaedics, Faculty of Medicine, Ramathibodi Hospital, Mahidol University, Bangkok 10400, Thailand; tulyapruek.tao@mahidol.ac.th (T.T.); pheeraphat.pho@mahidol.ac.th (P.P.); thepparat.kan@mahidol.ac.th (T.K.); 2 Chakri Naruebodindra Medical Institute, Faculty of Medicine, Ramathibodi Hospital, Mahidol University, Samut Prakan 10540, Thailand

There was an error in the original publication [1]. The authors identified an error concerning the time duration mentioned in the Results Section. The specific section in question is as follows:

From October 2020 to **February 2023**, 23 patients were enrolled, 10 males and 13 females, with an average age of 46 years. Approximately 50% of these patients had an injury in their dominant arm.

A correction has been made to the **Results Section**, **Paragraph 1**, as follows.

From October 2020 to **October 2022**, 23 patients were enrolled, 10 males and 13 females, with an average age of 46 years. Approximately 50% of these patients had an injury in their dominant arm. They were categorized into two groups based on their specific injuries: traumatic TFCC injury (8 participants) and distal end radius fracture with DRUJ instability (14 participants). In the period of study, no cases of Galeazzi fractures and Essex-Lopresti fractures were presented. Out of the total participants, 10 were randomly assigned to wear a sugar tong slab, while 13 were randomly assigned to wear a modified ulnar gutter slab. Only 1 participant was lost to follow-up, leaving 22 participants who were monitored until the end of the 6-week protocol period following surgery. No statistical difference was present in the demographic data between the two groups. (*p* < 0.05) (Table 1 and Figure 1).

The authors state that the scientific conclusions are unaffected. This correction was approved by the Academic Editor. The original publication has also been updated.
